# Influence of Direct Coronal Restoration Materials on the Fracture Resistance of Endodontically Treated Premolars: An In Vitro Study

**DOI:** 10.3390/dj12090294

**Published:** 2024-09-19

**Authors:** Georgiana Florentina Gheorghe, Ana Maria Cristina Țâncu, Oana Elena Amza, Ioana Suciu, Laura Iosif, Stanca Cuc, Ioan Petean, Marioara Moldovan, Bogdan Dimitriu

**Affiliations:** 1 Department of Endodontics, Faculty of Dentistry, Carol Davila University of Medicine and Pharmacy, 17-23 Calea Plevnei Street, 010232 Bucharest, Romania; georgiana.gheorghe@umfcd.ro (G.F.G.); ioana.suciu@umfcd.ro (I.S.); bogdan.dimitriu@umfcd.ro (B.D.); 2 Department of Prosthodontics, Faculty of Dentistry, Carol Davila University of Medicine and Pharmacy, 17-23 Calea Plevnei Street, 010232 Bucharest, Romania; anamaria.tancu@umfcd.ro; 3 Raluca Ripan Institute of Research in Chemistry, Babes-Bolyai University, 30 Fantanele Street, 400294 Cluj-Napoca, Romania; stanca.boboia@ubbcluj.ro (S.C.); marioara.moldovan@ubbcluj.ro (M.M.); 4 Faculty of Chemistry and Chemical Engineering, Babes-Bolyai University, 11 Arany Janos Street, 400084 Cluj-Napoca, Romania; ioan.petean@ubbcluj.ro

**Keywords:** root canal treatment, restoration material, endodontically treated tooth, premolar, fracture resistance, stereomicroscope, scanning electron microscopy

## Abstract

(1) Background: The long-term survival of an endodontically treated tooth depends on a successful root canal treatment as well as an adequate definitive coronal restoration. This study aimed to evaluate the strength of endodontically treated premolars with mesial–occlusal–distal (MOD) cavity preparation restored with different direct coronal restoration materials but from the same manufacturer against fracture. (2) Methods: sixty intact premolars were selected and placed into five groups (n = 12): G1—intact teeth, G2—endodontic treatment and unrestored MOD cavities, G3—endodontic treatment and MOD cavities restored with Tetric PowerFlow and Tetric EvoCeram, G4—endodontic treatment and MOD cavities restored with Multicore Flow and Tetric EvoCeram, and G5—endodontic treatment and MOD cavities restored with Multicore Flow. The specimens were subjected to an axial compression load at a speed of 1.6 mm/min and optically inspected before and after with a stereomicroscope. For each premolars group, the following data were recorded: the compression resistance, the compressive strength, and the maximum force supported. The microstructure of the samples after the compression test was analyzed using scanning electron microscopy (SEM). (3) Results: statistical analysis (ANOVA and Tukey test) showed that there was a statistically significant difference between G1 and the other groups. Even though there was no statistically significant difference between the restored groups, a better mechanical behavior was registered within the G3. (4) Conclusions: this in vitro study indicated that none of the materials used can lead to a higher or at least similar fracture resistance as the intact teeth. The coronal restoration only with nano-hybrid composites may lead to a higher therapeutic benefit for the fracture-susceptible premolars.

## 1. Introduction

The ideal technique for restoration of endodontically treated teeth (ETT) is still controversial because of their different biomechanical properties when compared to vital teeth. Fracture is more likely to happen to a tooth after a root canal treatment than to a vital one because of the loss of water content and anatomic structures [1]. The long-term survival of ETT is influenced by successful root canal treatment as well as the amount of remaining dentine. The root canal procedure is finished after an adequate definitive coronal restoration whose quality is important as it reduces the marginal microleakage [2]. Furthermore, the fracture resistance of ETT, particularly premolars, can vary significantly also depending on the approach used for root canal preparation. Minimally invasive endodontics (MIEs) consisting of minimal root canal preparations with an apical diameter ranging between 0.2 mm and 0.4 mm and a taper that is strictly below 6%, tends to preserve more of the natural tooth structure, which generally results in higher fracture resistance [3]. The smaller apical diameter and reduced taper help maintain the integrity of the tooth, reducing the likelihood of fractures. By maintaining more dentin, MIE helps in the better distribution of occlusal forces, which can lower the risk of root fractures. However, the smaller canal preparations may not be as effective in removing all the infected tissue and debris [4], potentially compromising the cleaning and shaping efficiency. On the other hand, larger taper instrumentation, in contrast, can contribute to deeper infected-tissue removal, achieving the appropriate irrigant penetration level but at the cost of increased risk of fractures. Despite these analyses, a recent significant report stated no clear evidence that taper differences significantly affect root fracture resistance [5].

Combined endodontic access cavity and mesial–occlusal–distal (MOD) cavity preparation increasingly reduce cusp deflection during function and fracture resistance [6,7]. Many clinicians have tried over the years to find the best option to restore an ETT. The treatment options for these teeth are multiple, varying from direct resin composites restoration to indirect restorations such as crowns, inlays, and onlays. Onlays are favored over crowns in MOD restorations due to their ability to conserve a greater amount of natural tooth structure while providing comparable strength and durability by encompassing the cusps, thereby enhancing the tooth’s structural integrity and aesthetic appeal [8]. Moreover, in comparison to inlays, onlays offer superior protection and support by covering the cusps, making them more suitable for extensive cavities that involve multiple surfaces [9].

Besides the popularity of onlays, some key aspects of their fracture resistance need to be highlighted. Thus, the type of the material used for onlays significantly affects the fracture resistance especially of the premolars. Studies have shown that CAD-CAM composite (resin nanoceramic) onlays tend to resist greater forces compared to ceramic restorations [10]. Although, incorporating fiber posts in teeth restored with ceramic onlays can improve the fracture resistance of endodontically treated premolars [11].

Even though the indirect restorations with cuspal coverage for the posterior ETT are still the elected choices due to their excellent aesthetics, the long-term higher success rate, minimal invasiveness, simplified procedures, and cost-effective ratio make direct restorations with resin composites a preferable approach. The main disadvantage of direct restorations is the polymerization shrinkage. To overcome this problem, the clinician can use the incremental layering technique by placing nano-hybrid composites with a thickness of 2 mm or bulk-fill composites placed with a thickness of 4 mm [12]. Further research indicates that endodontically treated premolars restored with conventional composite resin, bulk-fill composite, and ceramic inlays exhibit fracture resistance similar to that of sound teeth. Resin composites, known for their aesthetic and mechanical properties, are one of the most commonly used in such restorations. However, conventional composite resin restorations tend to have a higher prevalence of unrepairable fractures [13]. The application of various resin composites from a single manufacturer to improve the mechanical properties of premolars has received considerable scholarly attention in recent years. This focus arises from the necessity to enhance the longevity and durability of restorations in endodontically treated teeth, which are frequently more prone to fractures due to compromised structural integrity [14]. Although the combination of different types of composites from the same manufacturer is hypothesized to offer synergistic benefits, potentially enhancing the fracture resistance of the restored tooth, research in this area remains quite inconclusive.

For this study, premolar teeth were selected due to their increased susceptibility to distractive lateral forces compared to molars. We assessed the fracture resistance of different direct restorations, by combining different materials but from the same manufacturer, in endodontically treated premolars with mesial–occlusal–distal (MOD) cavities. The aim was to determine which association of materials from the same brand can offer a better result. Since composite materials are so popular, it is necessary to determine which one leads to better survival of an ETT and a successful outcome.

## 2. Materials and Methods

Sixty intact human mandibular and maxillary single-rooted premolars, extracted for reasons related to periodontal and orthodontic diseases, were used for this study in accordance with ethical guidelines of Carol Davila University of Medicine and Pharmacy Clinical Research Ethics Committee (ethical protocol PO-35-F-03b).

The inclusion criteria consisted in the lack of any enamel cracks and fractures, completely formed apices, no caries and no previous restorations, with all examinations being conducted under magnification and transillumination. Additionally, only those maxillary and mandibular premolars with similar anatomical crown heights were considered eligible. These measurements were taken using a digital caliper (Precise PS 7215, Burg Watcher, Wetter-Volmarstein an der Ruhr, Germany) from the occlusal surface to the cement enamel junction, as well as the mesial–distal and buccal–lingual dimensions at the occlusal surface.

After carefully removing calculus and soft tissue deposits with an ultrasonic scaler (D600, Guilin Woodpecker Medical Instruments, Guilin, China) and a hand scaler (Gracey curette SG 17/18, LM Dental, Parainen, Finland), artificial saliva was used to store the samples, at 4 degrees C until used. Preoperative radiographs were taken in two perpendicular directions (mesial–distal and buccal–lingual) to observe the root canal anatomy and determine the working length. The teeth were randomly divided into five groups for this study.

Using a high-speed diamond bur (Edenta AG, CH-9434 AU/SG, Gallen, Switzerland), under water cooling, standardized MOD cavities were prepared for all teeth, except the control group (intact teeth). The MOD cavities were prepared as follows: the buccal–lingual width was one third of the intercuspal distance and extended into the pulp chamber, the gingival margin was 1 mm above the cement enamel junction and the depth of the cavity was approximately 4 mm. For these measurements, we used a digital caliper (Precise PS 7215, Burg Watcher, Germany). The bur was replaced after every cavity preparation and the gingival floor was 1 mm above the cement enamel junction (CEJ). The internal lines were smoothed out. Endodontic access cavities were prepared using water-cooled diamond burs mounted on a fiber-optic high-speed handpiece (W&H Synea Fusion TG-98L RM, W&H Dentalwerk, Bürmoos, Austria). Working length determinations were performed with a size 10 K-file (Dentsply Sirona, Ballaigues, Switzerland) inserted up to the major apical foramen, and the root canals were instrumented with ProTaper Next instruments (Dentsply Sirona, Switzerland) up to size X3 (#30). During the endodontic treatment, 5.25% sodium hypochlorite (Chloraxid 5.25%, Cerkamed, Stalowa Wola, Poland) for irrigation was deposited using IrriFlex 30-G needles (Produits Dentaire SA, Vevey, Switzerland), 1 mL after each file was introduced into the canal. The root canals have been irrigated after being prepared, with 5 mL 17% ethylenediamine tetra acetic acid (Endo-Solution 17% EDTA, Cerkamed, Stalowa Wola, Poland), followed by a final rinse with 5 mL saline solution. Paper points were used to dry the canals that were filled then with gutta-percha cones (DiaDent, Burnaby, BC, Canada) and a polymeric calcium hydroxide endodontic sealer (Sealapex, Kerr Endodontics, Rastatt, Germany), using the cold lateral condensation technique. The coronal gutta-percha was removed with a heat-carrier and then compacted with a hand plugger (Dentsply Sirona, Switzerland). After the root canal filling, post-operative radiographs in two different dimensions (mesial–distal and buccal–lingual) were taken to evaluate the endodontic treatment.

To make certain that the procedure is uniform, the endodontic treatments were performed by one operator and the MOD preparations and restorations by another operator. According to the coronal restoration technique, the teeth were equally divided so that each group had the same number of upper and lower premolars, resulting in five groups of 12 teeth, as follows:

- Group 1: intact teeth used as negative control;
- Group 2: teeth with MOD cavities and endodontic treatment but left unrestored, used as positive control;
- Group 3: teeth with endodontic treatment and MOD cavities restored with Tetric Power Flow and Tetric EvoCeram. Before every restoration, a SuperMat Adapt Super Cap Matrix system (Kerr Hawe SA, Bioggio, Switzerland) was placed around the tooth structure. An etching gel containing 37% phosphoric acid (Total Etch, Ivoclar Vivadent AG, Schaan, Liechtenstein) was applied on the enamel and subsequently on the dentin and allowed a reaction time of 15 s. Afterwards, the phosphoric acid was thoroughly rinsed off with water spray and the tooth surfaces dried with oil-free air, avoiding excessive drying of the dentin. The cavity walls were bonded with Adhese Universal (Ivoclar Vivadent AC, Liechtenstein). After scrubbing the adhesive onto the tooth surface for 20 s, compressed air was used to disperse it, resulting in a glossy, immobile film layer, that was light-cured for 10 s with Bluephase G4 (Ivoclar Vivadent AG, Liechtenstein). Tetric PowerFlow (Ivoclar Vivadent AG, Liechtenstein) was applied in layers of 4 mm thickness, adapted to the cavity walls with a suitable instrument (probe) then light-cured for 10 s. For the final layer of 2 mm, Tetric EvoCeram was used with a suitable instrument and light-cured for 10 s. At the end, the finishing and polishing procedures were performed under water cooling once the matrix was removed;
- Group 4: teeth with endodontic treatment and MOD cavities restored with Multicore and Tetric EvoCeram. After etching and bonding, as was conducted in Group 3, MultiCore Flow (Ivoclar Vivadent AG, Liechtenstein) using the intra-oral tip was directly placed into the cavity and light-cured for 10 s. The final layer of 2 mm was filled in the same way as in Group 3, and the final steps were the same as for Group 3;
- Group 5: teeth with endodontic treatment and MOD cavities restored only with MultiCore Flow. The etching and bonding procedures were conducted as for Group 3, then MultiCore Flow filled the entire cavity and was light-cured, with the matrix removal being followed by the polishing procedure.

All the specimens were stored in artificial saliva at 37 degrees C for 1 week.

To simulate natural conditions, the teeth were covered with a thin layer (0.2–0.3 mm) of wax (DistriWax, Distrident Plus, Suceava, Romania) and inserted parallel with the long axis in autopolymerising acrylic resin cylinders (Duracryl Plus, SpofaDental, Kerr Corporation, Jicin, Czech Republic) up to 1 mm below the CEJ. Before the end of the resin setting time, the teeth were removed, the wax was cleaned, and a light-body silicone-based impression material (Oranwash L, Zhermack, Badia Polesine, Italy) was injected into the resin blocks, in order to simulate the periodontal ligament. Immediately, the wax-free teeth were inserted into the resin cylinders.

**Figure 1:**
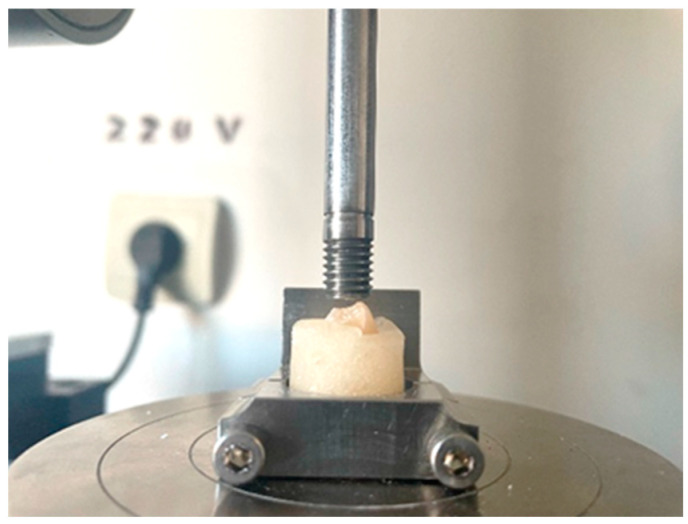
The compression test.

The samples were positioned in a mechanical material testing machine LR5K Plus that was controlled with a computer (Lloyd Instruments Ltd., Fareham, UK) and equipped with a maximum load of 5 k N. The continuous compressive force, measured in Newton (N), was preset at a rate of 1.6 mm/s and applied with a 7 mm diameter compressive head and a flat rectangular indenter, which compressed locally the desired area of the tooth surface until fracture occurred (Figure 1). Thus, the failure load was divided by the indenter surface, resulting in the compressive strength. The measurement itself was made via the testing machine software through the proper settings. The compressive strength and maximum force supported values were obtained from the computer software Nexygen (Ver 4, Lloyd Instruments, Bognor Regis, UK).

The samples were optically inspected and photographed before and after the compression test with a stereomicroscope (Stemi 2000-C, Carl Zeiss AC, Obercochen, Germany) at a 4× magnification. Then, the microstructure was investigated with a scanning electron microscope (SEM-Inspect S, FEI Company, Hillsboro, OR, USA) at a 50× and 500× magnification.

Means and standard deviations were determined for each group and data were statistically analyzed with one-way analysis of variance (ANOVA) to compare fracture resistance of the groups, followed by Tukey’s test, using the OriginPro 2024 program.

## 3. Results

The mean fracture resistance and standard deviation are presented in Table 1. They ranged from 1413.03 (243.68) N to 541.34 (276.01) N. The highest load at break was recorded for G1 (control group) and the lowest for G2 (unrestored teeth).

For the compression resistance, the obtained values are shown in Table 2.

**Figure 2:**
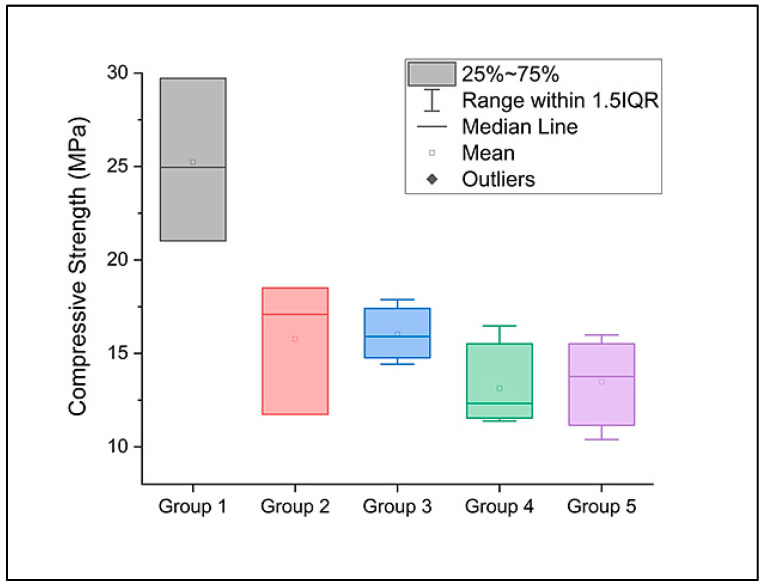
The compressive strength of the investigated premolars during the compression test.

At a significance level of 0.05, the compressive strength (Figure 2) values show statistically significant differences between the investigated groups (*p* = 6.24644 × 10^−4^). After the Tukey test, we found significant differences between the control group G1 and the rest of the groups.

For the maximum supported force, the recorded values are presented in Table 3.

**Figure 3:**
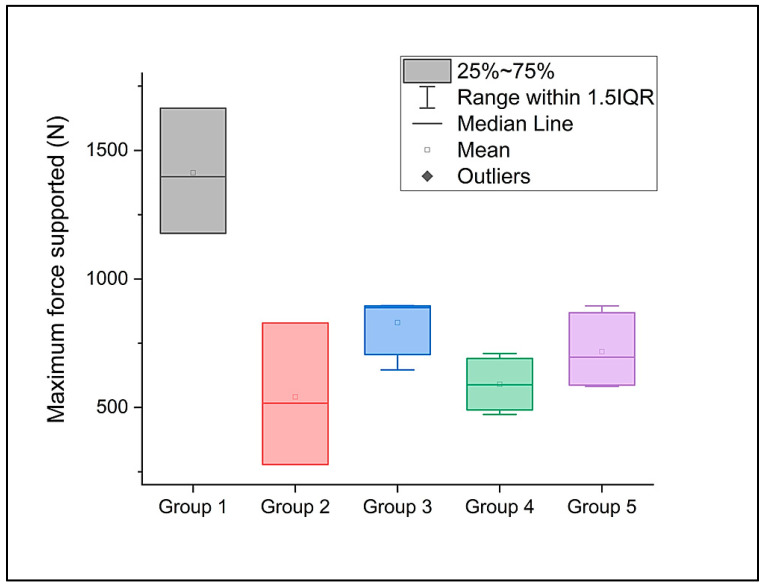
The maximum force supported by the investigated premolars during the compression test.

Statistically significant differences between the investigated groups can be seen in the maximum force supported during compression (Figure 3) at a significant level of 0.05, with the control group G1 being the only one with differences apart from the other groups.

**Figure 4:**
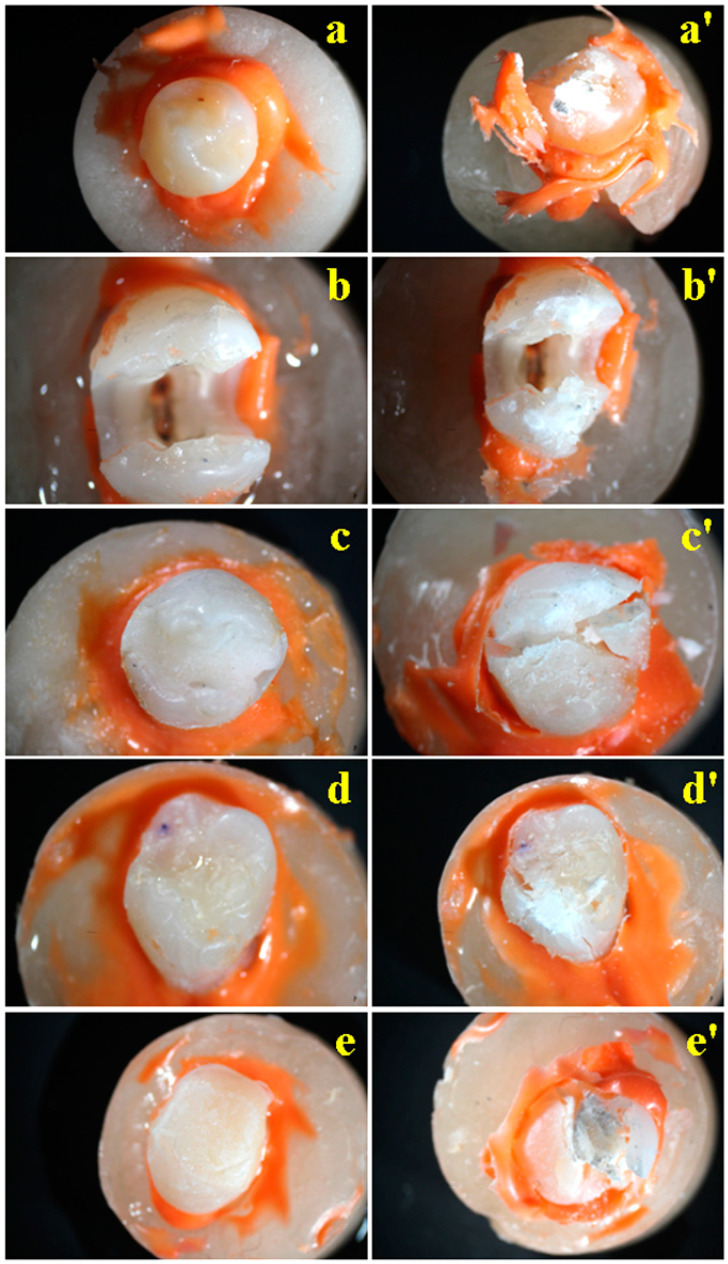
Photographs of the teeth samples before compression test: (**a**) Group 1, (**b**) Group 2, (**c**) Group 5, (**d**) Group 3, and (**e**) Group 4, and after the compression test: (**a′**) Group 1, (**b′**) Group 2, (**c′**) Group 5, (**d′**) Group 3, and (**e′**) Group 4.

Representative images of the macroscopic aspect of the samples are shown in Figure 4.

**Figure 5:**
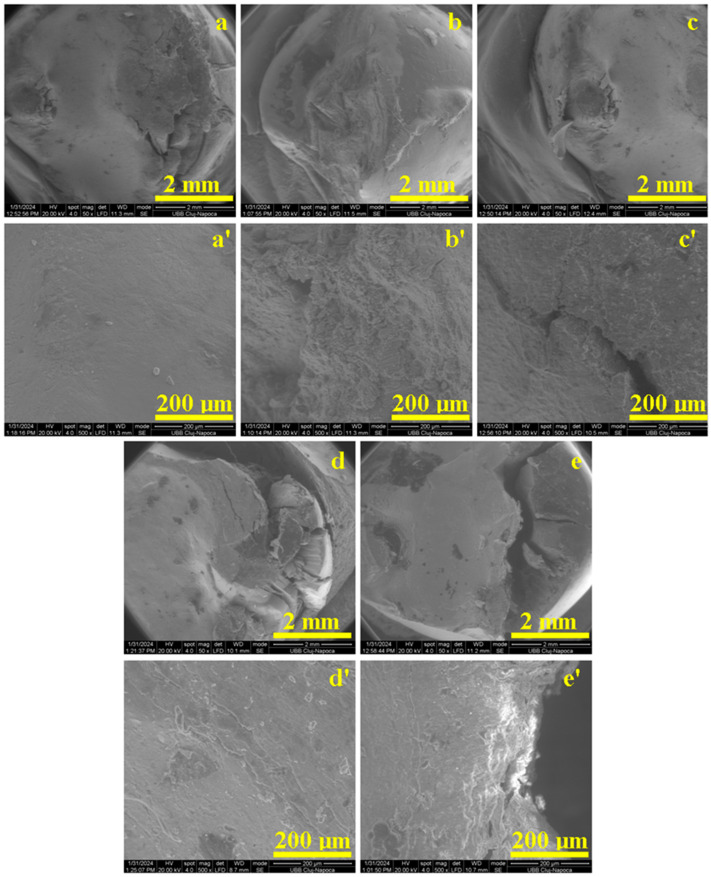
SEM images for the teeth samples’ microstructure after compression test observed at low magnification (50×): (**a**) Group 1, (**b**) Group 2, (**c**) Group 5, (**d**) Group 3, and (**e**) Group 4 and at high magnification (500×): (**a′**) Group 1, (**b′**) Group 2, (**c′**) Group 5, (**d′**) Group 3, and (**e′**) Group 4.

The sample’s microstructure after the compression test was investigated with SEM; some of the obtained results are presented in Figure 5.

## 4. Discussion

Endodontically treated premolars with mesial–occlusal–distal (MOD) cavities present a distinct challenge in restorative dentistry. The significantly compromised structure resulting from extensive cavity preparation necessitates the utilization of materials capable of restoring both function and strength [15]. Another reason for selecting premolars for this study was their cusp inclination, which makes them more susceptible to cusp fracture under occlusal force. In order to mimic a frequent clinical situation, we prepared MOD cavities that were restored using different materials but from the same manufacturer. Searching the literature, we found many studies regarding the restoration of MOD cavities in endodontically treated teeth, including premolars [16,17,18,19].

According to our study, premolars restored using adhesively bonded restorations have a lower fracture resistance compared to intact premolars. Furthermore, some other studies have reported higher fracture resistance for intact teeth [20,21,22,23,24].

We analyzed the most representative photographs taken before and after the compression test and the microstructure aspect of the same teeth after the compression test using SEM images. The healthy untreated tooth has a smooth and shiny surface with an intact and well-preserved enamel (Figure 4a). The morphologic aspect presents a prominent cusp with a spheroid form. The excessive tooth grinding (e.g., bruxism) was simulated by a small punch compression test oriented on the top of the tooth cusp by applying the force through a plane surface. The pressure is dissipating within the enamel structure which is damaged as observed in Figure 4a′. The SEM investigation reveals that the tooth receives a compression punch right on the top of the rounded cusp which is heavily affected (Figure 5a). The direct impact area is about 2 mm in size and preserves its cohesion and structure as can be seen in the high magnification detail of Figure 5a′. But the force is radially dissipated through the intact enamel surface and breaks the margins of the tooth which are situated outside of the punching die that are penetrated by uneven cracks that generate local debris containing particles with sizes in the range of 250–500 µm (Figure 5a). This result agrees with the studies of Plotino et al. [3] and Mergulhao et al. [13], where the intact teeth were found to have more restorable fractures than all the restored ones.

The unrestored tooth with endodontic treatment (Figure 4b) presents an intact enamel and dentin structure prepared to be filled with dental composites. The applied force affects the lateral sides of cavity walls causing several cracks on the top layers with some breaking debris (Figure 4b′). The microstructure of this tooth, after the compression test, was observed with SEM, revealing the side failure under compressive force that is laterally ruined (Figure 5b). The force is propagated through the dentin at an angle of about 45° where the hydroxyapatite unit’s network was fractured (Figure 5b′), proving an intra-crystalline failure. This is caused by the lack of mechanical support on the side cavity walls. Filling it with composite material will bring significant support to avoid the dentin intra-crystalline failure.

The general aspect of a tooth restored with Multicore Flow composite feature a uniform and shining appearance having great resemblance to a healthy tooth (Figure 4c). There is also a rounded cusp of the tooth that is subjected to compressive force, which breaks the tooth in two parts through the composite layer, indicating its failure while the natural tooth components (e.g., enamel and dentin) remain relatively intact (Figure 4c′). The Multicore restoration macroscopic aspect reveals the composite failure through its bulk. Therefore, the SEM observation focused on the near-proximal impact area in the center of the SEM image in Figure 5c. Thus, the die punch streaks a local peak of about 1 µm diameter absorbing the solicitation force and dissipating it through the composite microstructure. Figure 5c’s high magnification detail reveals the crack propagation through some fine pores in a size range of about 50–150 µm with an elongated shape in the molding direction. The material situated between these pores is locally stressed causing sudden delaminating of the filler particles that further allows for crack propagation. MultiCore Flow is a dual-cure resin composite used for core build-up in vital and non-vital teeth. It was developed to overcome the limitations of light-cured materials. Because it can be polymerized by both light and self-activation, it is a good alternative for restoring deep cavities with a bulk technique [25]. In the present study, we wanted to see how this material can withstand occlusal forces, considering that many patients do not return to the practice for an indirect restoration after the core build-up of the tooth. The sudden delaminating of the filler particles may be because of lower viscosity of the uncured MultiCore Flow.

The restoration performed with Tetric PowerFlow and Tetric Evoceram has a more flattened aspect like a molar surface (Figure 4d). The pressure exerted during the compression test is supported by the restoration composite that remains almost intact. Thus, it dissipates the compressive force on the natural tooth structures which are overstressed. This fact finally causes the composite—enamel and composite—and dentin junction failure. The crack is deeply propagated within the hybrid layer (Figure 4d′).

The tooth restored with Multicore and Tetric EvoCeram has a uniform and compact surface with a bright and shiny aspect that deals with a high-quality treatment (Figure 4e). The compressive force punches the composite cusp of the restoration which exhibit resistance under solicitation, dissipating the force to the enamel and dentin through the hybrid layer. The force within the hybrid layer causes its failure and generates a macroscopic crack as is visible in Figure 4e′.

Both Tetric composite samples present a compact and dense microstructure in the right side of the SEM images in Figure 5d,e, while the hybrid layer totally failed, and the crack is filled with collateral debris detached from the enamel side. The cohesion of Tetric PowerFlow is proved by the high magnification detail in Figure 5d′, where the flow lines are still visible. There are some bigger filler particles of about 100 µm associated with nano-filer that are very well embedded on the polymer matrix. Such compact microstructure is very resistant to punching shock. The Tetric composite (Figure 5e′) presents more refined filler particles very well embedded in the polymer matrix, and the right side of the image evidences the hybrid layer failure being completely detached from the tooth structure.

The overall aspects allow us to observe that the Multicore composite is very sensitive to bruxism-like solicitation causing mostly internal failure, while the Tetric composite resists very well the bruxism solicitations, but the failure occurs within the hybrid layer.

Regarding the dual-cure resin composites applied in the bulk technique, despite their mechanical long-term success being questionable, some studies have reported good results. [26,27], and their popularity has risen due to their promise of reducing chair time and placement of up to 4–5 mm in large cavities. Direct composite restorations are popular because of their aesthetic and physical properties, simple application, and the fact that they can be placed in a single session. Our findings showed that endodontically treated premolars that were restored with composite resins placed in increments and bulk did not exhibit significantly different mean fracture resistance compared to other studies [22,28,29,30].

To standardize the preparation procedures, we utilized only caries-free premolars [31,32]. In a clinical setting, we encounter teeth with thinner walls, less dentin, and irregular margins, so they should be restored before starting the endodontic treatment.

The compression values for restored teeth can vary depending on factors such as the type of material used for restoration (e.g., composite resin, amalgam, or ceramic), the quality of the restoration, and the condition of the tooth itself. Additionally, the clinical performance of a restoration depends not only on its compressive strength but also on other factors such as bonding to the tooth structure, wear resistance, and resistance to fracture under various loading conditions.

The clinical significance of our research lies in the observation that using nano-hybrid composites for the coronal restoration of endodontically treated premolars may improve their fracture resistance. This can be attributed to the fact that nano-hybrid composites combine the advantages of both microfill and hybrid composites, providing superior mechanical properties and aesthetics. Additionally, nano-hybrid composites offer excellent adhesion to the tooth structure, which helps maintain marginal integrity and enhances the overall durability of the restoration [33]. In our study, we simulated intraoral loads, applying them only vertically and not laterally, which are forces that may occur during clenching. Also, to evaluate the performance of direct restorations, additional tests like finite element analysis and the tensile test are required along with a compressive load test.

Our study was exploratory in nature, aiming to identify potential trends regarding the combination of resin composites as direct restoration materials in terms of a higher fracture resistance of ETT and to generate hypotheses for future research. As such, a power analysis was not conducted, and the sample size was determined based on practical considerations. Another methodological limitation was that the research utilized freshly extracted, intact teeth. In typical clinical scenarios, an ETT would likely have been restored prior to endodontic therapy. Consequently, the coronal walls might exhibit asymmetrical contours and varying thicknesses, and the intact dentin would be thinner than that of the premolars we investigated. Another aspect was the absence of a control group that would allow for a comparative analysis between the fracture resistance of ETT restored with onlays for cusp protection and those restored using combinations of two different resin composites. Future research should focus on this comparison to determine the most effective restorative approach for enhancing the structural integrity and longevity of endodontically treated premolars. Due to this study’s limitations, more clinical investigations are necessary to determine which direct composite restoration should be used as an efficient and low-cost procedure for endodontically treated premolars.

## 5. Conclusions

Endodontically treated premolars restored with different composite materials showed a lower fracture resistance than intact teeth. Our in vitro study showed that MOD cavity preparations significantly decreased the fracture resistance of endodontically treated premolars. Still, none of the combinations of tested restoration materials could replace the primary fracture resistance. Even though there was not a statistically significant difference, better results were obtained by combining a flowable and a compacted nano composite.

Due to extensive use of direct restoration, it is important for practitioners to gain knowledge about what materials and techniques can improve the longevity of teeth with endodontic treatment.

## Figures and Tables

**Table 1:** The fracture resistance of the investigated premolars during the compression.

Group		Compressive Strength (MPa)	Maximum Force Supported (N)
1	Mean	25.23273	1413.03281
	Standard deviation	4.35149	243.68338
2	Mean	15.77553	541.34753
	Standard deviation	3.56558	276.01193
3	Mean	276.01193	829.9073
	Standard deviation	276.01193	122.60253
4	Mean	122.60253	590.00804
	Standard deviation	2.28742	103.32499
5	Mean	13.47568	717.00178
	Standard deviation	2.32245	150.90633

**Table 2:** The compression strength of the investigated premolars during the compression test (ANOVA).

	Sum of Squares	Mean Square	F Value	Prob > F
Model	312.70978	78.17744	10.04156	6.24644 × 10^−4^
Error	101.21002	7.78539		
Total	413.9198			

**Table 3:** The maximum force supported by the investigated premolars during the compression test (ANOVA).

	Sum of Squares	Mean Square	F Value	Prob > F
Model	1,535,643.58363	383,910.89591	11.98083	2.66559 × 10^−4^
Error	416,568.81433	32,043.75495		
Total	1,952,212.39795			

## Data Availability

Data are available on request from the corresponding authors.
